# Re-emergence of H3N2 strains carrying potential neutralizing mutations at the N-linked glycosylation site at the hemagglutinin head, post the 2009 H1N1 pandemic

**DOI:** 10.1186/s12879-016-1738-1

**Published:** 2016-08-08

**Authors:** Hiroshi Ushirogawa, Tadasuke Naito, Hirotoshi Tokunaga, Toshihiro Tanaka, Takashi Nakano, Kihei Terada, Masanobu Ohuchi, Mineki Saito

**Affiliations:** 1 Department of Microbiology, Kawasaki Medical School, 577 Matsushima, Kurashiki, Okayama 701-0192 Japan; 2 Department of Hematology, Kawasaki Medical School, 577 Matsushima, Kurashiki, Okayama 701-0192 Japan; 3 Department of Pediatrics, Shizuoka Kosei Hospital, 23 Kitaban-cho, Aoi-ku, Shizuoka 420-8623 Japan; 4 Department of Pediatrics, Kawasaki Medical School, 577 Matsushima, Kurashiki, Okayama 701-0192 Japan

**Keywords:** Influenza A, H3N2, N-glycosylation, HA globular head, Escape mutation

## Abstract

**Background:**

Seasonally prevalent H1N1 and H3N2 influenza A viruses have evolved by antigenic drift; this evolution has resulted in the acquisition of asparagine (N)-linked glycosylation sites (NGSs) in the globular head of hemagglutinin (HA), thereby affecting the antigenic and receptor-binding properties, as well as virulence. An epidemiological survey indicated that although the traditional seasonal H1N1 strain had disappeared, H3N2 became predominant again in the seasons (2010–11 and 2011–12) immediately following the H1N1 pandemic of 2009. Interestingly, although the 2009 pandemic H1N1 strain (H1N1pdm09) lacks additional NGSs, clinically isolated H3N2 strains obtained during these seasons gained N (Asn) residues at positions 45 and 144 of HA that forms additional NGSs.

**Methods:**

To investigate whether these NGSs are associated with re-emergence of H3N2 within the subtype, we tested the effect of amino acid substitutions on neutralizing activity by using the antisera raised against H3N2 strains with or without additional NGSs. Furthermore, because the N residue at position 144 of HA was identified as the site of mismatch between the vaccine and epidemic strains of 2011–2012, we generated mutant viruses by reverse genetics and tested the functional importance of this particular NGS for antibody-mediated neutralization by intranasal inoculation of mice.

**Results:**

The results indicated that amino acid substitution at residue 144 significantly affected neutralization activity, acting as an escape mutation.

**Conclusions:**

Our data suggest that the newly acquired NGSs in the HA globular head may play an important role in the re-emergence of endemic seasonal H3N2 strain by aiding the escape from humoral immunity.

## Background

Although influenza A viruses (IAVs) infect many avian and mammalian species, only three subtypes (H1N1, H2N2, and H3N2) infect and transmit efficiently among humans [1]. Viral hemagglutinin (HA) is the major antigenic glycoprotein responsible for binding the virus to cells, and it is, therefore, the major target of neutralizing antibodies. Indeed, the accumulation of amino acid substitutions, known as “antigenic drift,” which allows the virus to escape from the neutralizing antibody response, frequently occurs in HA [2–4]. The attachment of an oligosaccharide to the N-glycosylation sites (NGSs) in the globular head region of HA via N-glycosylation sequons (i.e., Asn-X-Ser/Thr, where X is any amino acid, except Pro) contributes to the escape of viruses from host immune response [5]. The number of NGSs in the globular head region of H3N2 virus HA has increased during circulation in the human population [6–9], and most of the currently circulating H3N2 viruses have six (Asn residues 63, 122, 126, 133, 165, and 246) or seven (Asn residues 63, 122, 126, 133, 144, 165, and 246) NGSs. When the H3N2 virus first emerged in the human population in 1968, there were only two NGSs, at amino acid positions 81 and 165, in the globular head region of HA. Thereafter, the appearance or disappearance of glycosyl chains in the globular head has been reported to occur naturally during the antigenic drift of H3N2 from 1968 to 1975 [2, 6, 10]. Glycosylation of HA has been shown to modulate the sensitivity of H3N2 viruses to innate proteins in airway secretion, the virulence properties of the strain [11], and the replication of viruses in the respiratory tract of ferrets [12].

Meanwhile, it is well known that H3N2 is one of the major subtypes of IAV that circulate in humans after the Hong Kong flu pandemic of 1968 [1]. According to the National Institute of Infectious Diseases in Japan (http://www.nih.go.jp/niid/en/), although the traditional seasonal influenza H1N1 virus caused the epidemic in 2008, this virus disappeared after the 2009 pandemic of H1N1 virus (H1N1pdm09), and H1N1pdm09 appears to have replaced the traditional H1N1 virus. This observation is supported by the fact that the rates of resistance to oseltamivir was 100 % among the H1N1 viruses circulating in Japan in the 2008–09 season, but suddenly dropped to 0.5 % in the 2010–11 season after the 2009 pandemic. However, the H1N1pdm09 lacks additional NGSs in the globular head region of HA. In contrast to the traditional H1N1, H3N2 became predominant again in Japan in the seasons (2010–11 and 2011–12) immediately following the 2009 pandemic of H1N1. These findings prompted us to investigate whether the re-emergence of H3N2 is due to a change in the HA within this subtype that escapes immunity from the prior H3N2 virus.

In this study, we therefore conducted a retrospective analysis of the amino acid substitutions of the HA globular head observed in seasonal H3N2 viruses during two consecutive seasons (2010–11 and 2011–12) immediately after the 2009 pandemic. Furthermore, using multiple approaches, we examined whether the presence and/or absence of N-linked glycans in HA affects viral neutralization, and in this manner, the re-emergence of H3N2.

## Methods

### Retrospective database analysis

To elucidate the annual epidemics of seasonal influenza virus after the 2009 pandemic, we referred to the websites of National Institute of Infectious Diseases in Japan (http://www.nih.go.jp/niid/en/) and the Influenza Virus Resource of the National Center for Biotechnology Information (NCBI) (http://www.ncbi.nlm.nih.gov/genomes/FLU/FLU.html) [13].

### Viruses and cells

Clinical specimens from patients with confirmed IAV H3N2 infection were obtained during two consecutive seasons (2010–11 and 2011–12) immediately after the 2009 pandemic of H1N1pdm09. Each specimen was propagated once in Madin-Darby canine kidney cells overexpressing α-2,6-sialyltransferase (MDCK-SIAT1 cells; DS Pharma Biomedical, Japan) in Eagle’s minimum essential medium (MEM) supplemented with 10 % fetal calf serum and 1 μg/ml acetylated trypsin (Sigma, Japan). For subsequent experiments, we chose four stored H3N2 isolates (virus stocks) from each season, in the order in which they were obtained, while also making sure that sufficient quantities of purified virus were available. All samples were obtained from patients who had not received any medication prior to sample collection. Four samples (A/Okayama/2/11, A/Okayama/3/11, A/Okayama/4/11, and A/Okayama/5/11) were isolated in the Okayama prefecture in the 2010–11 season and four samples (A/Shizuoka/10/12, A/Shizuoka/23/12, A/Shizuoka/24/12, and A/Shizuoka/26/12) were isolated in the Shizuoka prefecture in the 2011–12 season. All samples were stored in liquid nitrogen until use.

### RT-PCR and sequence analysis

Viral suspensions propagated in MDCK-SIAT1 cells were centrifuged at 10,000 × *g* for 90 min at 4 °C, and viral RNA was extracted from the precipitate by using ISOGEN (Wako Chemicals, Japan). Subsequently, cDNA was synthesized from RNA by using the Omniscript RT Kit (QIAGEN, Germany) and reverse transcription-polymerase chain reaction (RT-PCR) was performed using Pyrobest polymerase (Takara, Japan) with the following primers: forward, 5’-TAA TTC TAT TAA CCA TGA AG-3’; reverse, 5’-TTT TTA ATT AAT GCA CTC AAA TGC-3’. The PCR products were subjected to agarose gel electrophoresis, and the specific bands were excised from the gel and purified using a QIAquick Gel Extraction Kit (QIAGEN, Germany). The purified PCR products were subjected to direct sequencing.

### Generation of recombinant viruses

RNA polymerase I-driven expression plasmid (pPolI) expressing each gene segment of WSN and pCAGGS plasmids expressing the WSN viral proteins, PA, PB1, PB2, and NP, were kindly provided by Prof. Yoshihiro Kawaoka (University of Wisconsin). The cDNA of HA gene of A/Okayama/6/01 (H3N2) was prepared by RT-PCR and cloned into the pPolI vector designated as pPolI-Oka/6/01-wt (code named H3-0) in our previous study [14]. For constructing HA mutant plasmid lacking glycosylation of Lys144 residue, which is designated as pPolI-Oka/6/01-mutant (code named H3-1), a single amino acid substitution, from Ser to Ala, at residue 146 was introduced into the pPolI-Oka/6/01-wt (H3-0) plasmid by using the following primers: 5’-AGA TCT AAT AAA GCT TTC TTT AGT AGA-3’ and 5’- TCT ACT AAA GAA AGC TTT ATT AGA TCT-3’. 293T cells were prepared as half-to-three-fourth confluence on the wells of 6-well cell culture plate for plasmid transfection. pPolI-Oka/6/01 (H3-0 or H3-1) and other pPolI plasmids encoding the vRNA of seven internal genes derived from WSN were transfected together with the pCAGGS plasmid into 293T cells by TransIT-293 Transfection Reagent (Minus Bio, USA), according to the manufacturer’s instructions. Transfected 293T was incubated at 37 °C in OPTI-MEM and the supernatant was harvested at 48 h post transfection. MDCK cells were inoculated with the collected supernatant to amplify the rescued viruses.

### Preparation of antisera

After sequence analysis of the clinically isolated viruses, we chose three types of viruses for the production of polyclonal antisera in guinea pigs: A/Okayama/2/11 (Oka/2) as the 144K type, A/Shizuoka/23/12 (Sk/23) as the 144N type, and A/Shizuoka/26/12 (Sk/26) as the 144N/45N type (Table 1). These viruses were propagated in MDCK-SIAT1 cells and concentrated. Subsequently, 4-week-old naïve female guinea pigs (Hartley strain; Japan SLC, Hamamatsu, Japan) were intraperitoneally primed and boosted with each concentrated virus suspension mixed with an adjuvant (TiterMax Gold; CytRx Co., USA) at 2-week intervals. Finally, the antisera were prepared from the whole blood and stored at −80 °C until use. The sera from recovered virus-infected mice were also stocked at −80 °C. All animal experiments were approved by the Institutional Animal Care and Research Advisory Committee of Kawasaki Medical School, prior to initiation of the study.

**Table 1:** Glycosylation sites in the hemagglutinin (HA) of H3N2 viruses isolated from clinical specimens

Virus	Donor		Glycosylation sites in HA							
	Age	Sex	45	63	122	126	133	144	165	246
2010–11 season										
A/Okayama/2/11 (Oka/2)	4	F	SSS	NCT	NES	NWT	NGT	KNS	NVT	NST
A/Okayama/3/11	5	M	SSS	NCT	NES	NWT	NGT	NNS	NVT	NST
A/Okayama/4/11	6	M	SSS	NCT	NES	NWT	NGT	KNS	NVT	NST
A/Okayama/5/11	12	F	SSS	NCT	NES	NWT	NGT	KNS	NVT	NST
2011–12 season										
A/Shizuoka/10/12	4	M	NSS	NCT	NES	NWT	NGT	NNS	NVT	NST
A/Shizuoka/23/12 (Sk/23)	2	F	SPS	NCT	NES	NWT	NGT	NNS	NVT	NST
A/Shizuoka/24/12	5	M	NSS	NCT	NES	NWT	NGT	NNS	NVT	NST
A/Shizuoka/26/12 (Sk/26)	5	M	NSS	NCT	NES	NWT	NGT	NNS	NVT	NST

Residue 45 is located in the stem of HA, and residues 63, 122, 126, 133, 144, 165, and 246 are located in the globular head of HA

Oka/2, Sk/23, and Sk/26 were used as representative 144K, 144N, and 45N/144N type viruses, respectively, to obtain neutralizing antibodies

All samples were obtained from patients who had not received any medication prior to sample collection. Four samples (A/Okayama/2/11, A/Okayama/3/11, A/Okayama/4/11, and A/Okayama/5/11) were isolated in the Okayama prefecture in the 2010–11 season and four samples (A/Shizuoka/10/12, A/Shizuoka/23/12, A/Shizuoka/24/12, and A/Shizuoka/26/12) were isolated in the Shizuoka prefecture in the 2011–12 season

### Hemagglutination inhibition (HI) assay

HA inhibition (HI) tests were performed for antigenic characterization of the isolates. Two-fold serial dilution lines of receptor-destroying enzyme (RDE)-treated antiserum in a volume of 25 μl were prepared with phosphate-buffered saline (PBS), and 25 μl of the virus antigen adjusted to 8 HA units/50 μl was added to each well. After 60-min incubation at room temperature, 50 μl of 0.5 % chicken erythrocytes in PBS was added to each well. After additional incubation for 45 min, the wells showing a block of HA were considered HI positive; the HI value was determined as the reciprocal of the dilution that was effective for HI.

### Microneutralization assay

The procedure for influenza virus microneutralization assay was modified from that listed in the WHO Manual on Animal Influenza Diagnosis and Surveillance 2002 version (http://www.who.int/csr/resources/publications/influenza/whocdscsrncs20025.pdf). Namely, three-fold serial dilution lines of the receptor-destroying enzyme-treated antiserum in a volume of 25 μl were prepared with virus growth medium (MEM containing 2 mM L-glutamine, 1× MEM amino acids, 1× MEM vitamin, 10 mM HEPES, and 0.2 % bovine albumin). Next, 20 μl of the virus antigen adjusted to 8 HA units/50 μl was added to each well. After 30-min incubation at room temperature, the antigen-virus mixtures were transferred into MDCK cells seeded in 96-well plates containing 2.5 μg/ml of tolylsulfonyl phenylalanyl chloromethyl ketone-trypsin (Sigma, Japan). After four days of incubation, the viral cytopathic effect was observed under an inverted microscope or by cell staining using amide black 10B.

### Plaque reduction neutralization test

A total of 100 plaque-forming units (PFU) of each viral suspension were incubated with the same volume of 10-fold serial dilutions of antiserum in PBS at 37 °C. After 1-h incubation, each sample was subjected to plaque assay in MDCK-SIAT1 cells and indirect immunostaining as previously described [15].

### Statistical analysis

The Mann-Whitney *U* test was used to compare data between two groups. Values of p < 0.05 were considered statistically significant.

## Results

### Re-emergence of epidemic H3N2 influenza viruses after the 2009 H1N1 pandemic

**Fig. 1:**
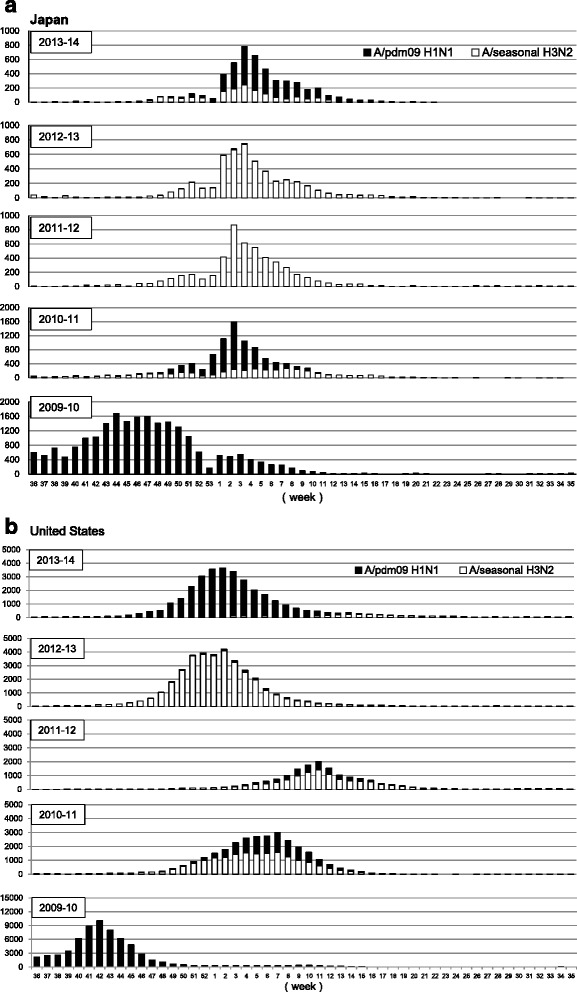
Re-emergence of epidemic H3N2 strains post 2009 H1N1 pandemic. We referred to the websites of National Institute of Infectious Diseases in Japan (http://www.nih.go.jp/niid/en/) and the Influenza Virus Resource of the National Center for Biotechnology Information (NCBI) (http://www.ncbi.nlm.nih.gov/genomes/FLU/FLU.html) to obtain the annual epidemiological data for seasonal influenza virus after the 2009 pandemic. H3N2 became predominant again in the seasons immediately following the 2009 pandemic of H1N1 (2010–11 and 2011–12), although the H1N1pdm09 virus became endemic again in the 2013–14 season both in **a** Japan and **b** United States

In order to characterize the annual epidemics of seasonal influenza virus after the 2009 pandemic, we first referred to the websites of the National Institute of Infectious Diseases in Japan (http://www.nih.go.jp/niid/en/) and the Influenza Virus Resource of NCBI (http://www.ncbi.nlm.nih.gov/genomes/FLU/FLU.html). As shown in Fig. 1a, a summary of the weekly reports of influenza virus isolation/detection in Japan indicated that H3N2 became predominant again in the seasons immediately following the 2009 pandemic of H1N1. As shown in Fig. 1b, a similar finding was observed in United States, which is geographically far from Japan and where extensive data were available.

### H3N2 influenza viruses that re-emerged after the 2009 H1N1 pandemic have the N-glycosylation-associated amino acid residue 144 in the HA globular head

We next investigated whether amino acid substitutions in the NGSs of the globular head region of HA are associated with the re-emergence of H3N2 within the subtype after the 2009 H1N1 pandemic. We retrieved from the Influenza Virus Resource of NCBI, the amino acid sequences for the HA genes of the seasonal H3N2 viruses from the two consecutive seasons (2010–11 and 2011–12) following the 2009 pandemic in Japan. The data of amino acid alterations at the glycosylation sites in the globular head and stem regions of HA are summarized in Table 2. As can be seen from this compilation, only three of the 11 virus isolates (27.3 %) from 2010–11 season carry an N residue that forms an additional NGS at residue 144 of HA, whereas almost all the strains (97.9 %) from the subsequent season of 2011–12 had this N residue.

**Table 2:** Glycosylation sites in the hemagglutinin (HA) of H3N2 strains isolated from the clinical specimens included in the database

Virus	Glycosylation sites in HA							
	45	63	122	126	133	144	165	246
2010–11 season (11 samples)								
8 samples (72.7 %)	SSS	NCT	NES	NWT	NGT	KNS	NVT	NST
3 samples (27.3 %)	SSS	NCT	NES	NWT	NGT	NNS	NVT	NST
2011–12 season (47 samples)								
32 samples (68.0 %)	SSS	NCT	NES	NWT	NGT	NSS	NVT	NST
4 samples (8.5 %)	NSS	NCT	NES	NWS	NGT	NSS	NVT	NST
2 samples (4.3 %)	NSS	NCT	NES	NWT	NGT	NSS	NVT	NST
1 sample (2.1 %)	NSS	NCT	NES	NWT	NGT	NNS	NVT	NST
1 sample (2.1 %)	SSS	NCT	NES	NWT	NGT	NNS	NVT	NST
1 sample (2.1 %)	SSS	NCT	NES	NWT	NGT	NSS	NVT	TST
1 sample (2.1 %)	SSS	NCT	NES	NWT	NGT	DSS	NVT	NST
1 sample (2.1 %)	NSS	NCT	NES	NWT	NGT	NSS	NVT	NST
1 sample (2.1 %)	SSS	NCT	NES	NWT	NGT	NNS	NVT	NST
1 sample (2.1 %)	SST	NCT	NES	NWT	NGT	NSS	NVT	NST
1 sample (2.1 %)	NSS	NCT	NES	NWS	NGT	NNS	NVT	NST
1 sample (2.1 %)	NSS	NCT	NES	NWS	NGT	NNS	NVT	NST
1 sample (2.1 %)	SSS	NCT	NES	NWS	NGT	NNS	NVT	NST

Residue 45 is located in the stem of HA, and residues 63, 122, 126, 133, 144, 165, and 246 are located in the globular head of HA

### Variation in the N-glycosylation-associated amino acid residue 144 in the HA globular head

**Fig. 2:**
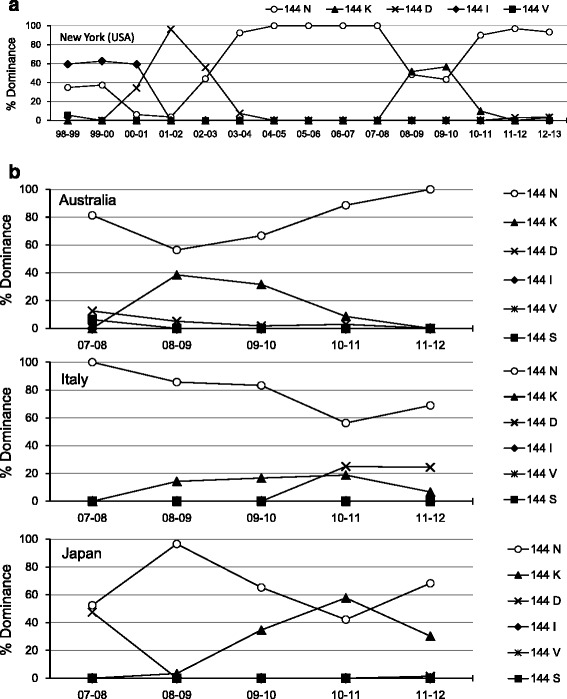
Fluctuation of the N-linked glycosylation-associated amino acid residue 144 in the globular head of hemagglutinin (HA). To analyze the characteristic features of the potential N-linked glycosylation at residue 144 of H3N2 HA globular head, we performed database search using the Influenza Virus Resource of NCBI (http://www.ncbi.nlm.nih.gov/genomes/FLU/FLU.html). There were frequent longitudinal alterations of the amino acid residue 144 of HA, and the dominant virus strain was frequently replaced. **a** New York (USA) **b** Australia, Italy and Japan

Further, to analyze the characteristic features of the potential NGS at residue 144 of H3N2 HA globular head, we performed database search using the Influenza Virus Resource of NCBI (http://www.ncbi.nlm.nih.gov/genomes/FLU/FLU.html) (Fig. 2). We first focused on the virus strains isolated in New York from 1998 to 2013, for which, most extensive data were available. The regions of ambiguous alignment were excluded. As shown in Fig. 2a, there were frequent longitudinal alterations of the amino acid residue 144 of HA; the replacement was more frequent in the dominant virus strain. For example, when 144N (gain of NGS) variant appeared as the dominant strain, the others (144D, 144K, 144I, and 144 V; loss of NGS) disappeared. Interestingly, similar variation of NGS at residue 144 of HA globular head was observed in countries across both the hemispheres, in Australia, Italy and Japan (Fig. 2b).

### Isolation of H3N2 influenza viruses with N-glycosylation-associated amino acid residue 144 in the HA globular head that re-emerged after the 2009 H1N1 pandemic

To characterize the potential NGS at residue 144 of HA globular head experimentally, we isolated viral strains from pediatric respiratory secretions during the 2010–11 and 2011–12 seasons in Japan and performed sequencing analysis of the HA region, using four clinical isolates from each season, eight strains in total (Table 1). Consistent with the epidemiological data, three of the four isolates from the 2010–11 season had a K residue at position 144 of HA (A/Okayama/2/11, A/Okayama/4/11, and A/Okayama/5/11), while all four isolates from the next season (2011–12) had an N residue in the same position (A/Shizuoka/10/12, A/Shizuoka/23/12, A/Shizuoka/24/12, and A/Shizuoka/26/12) (Table 1). Moreover, in the 2011–12 season, three of the four strains showed S45N substitution, which forms an additional NGS, and one showed S46P substitution (A/Shizuoka/23/12). To summarize, clinical isolates of H3N2 strains from the 2011–12 season gained N (Asn) residues at positions 45 and 144 of HA that form additional NGSs.

### Effect of amino acid substitutions at position 144 of the HA globular head on viral neutralization, as evaluated by HI and microneutralization assays

To determine whether amino acid substitutions in the HA stem and globular head regions (residues 45 and 144, respectively) affect the neutralizing activity of antisera, we raised guinea pig antisera against three clinically isolated viral strains in our laboratory (Oka/2 as the 144K type, Sk/23 as the 144N type, and Sk/26 as the 45N/144N type) (Table 1). The reactivity of each of the three antisera with each of the three viral antigens was examined by both HI and microneutralization assays (Table 3). As shown in Table 3, the homologous combination of Oka/2 antigen and antiserum showed HI and microneutralization titers of 160 and 270, respectively. The heterologous combination of Sk/23 antigen and anti-Oka/2 antiserum showed HI and microneutralization titers of 40 and 90, and Sk/26 antigen and anti-Oka/2 antiserum showed HI and microneutralization titers of 80 and 90, respectively, which are significantly lower than those of the homologous combination. In contrast, the heterologous combinations of Oka/2 antigen and anti-Sk/23 antiserum, and Oka/2 antigen and anti-Sk/26 antiserum showed substantial HI and microneutralization titers (320 and 270 for anti-Sk/23 antiserum, 320 and 405 for anti-Sk/26 antiserum) compared to the homologous combinations of Sk/23 or Sk/26 antigen and antiserum (160 and 90 for anti-Sk/23 antiserum, 640 and 270 for anti-Sk/26 antiserum) (Table 3). These results indicate that the antiserum against the 2010–11 season virus (Oka/2 HA-144K type) has reduced neutralizing activity against the 2011–12 season viruses (HA-144N types Sk/23 and Sk/26), whereas the antiserum against the 2011–12 season viruses (HA-144N types Sk/23 and Sk/26) has good neutralizing activity against the 2010–11 season virus (Oka/2 HA-144K type). This suggests the possible involvement of amino acid substitution at residue 144 of HA.

**Table 3:** Hemagglutination inhibition and neutralization reactivity by the H3N2 strains isolated during the 2010–11 and 2011–12 seasons

	Antibody titer in guinea pig serum					
	Oka/2 144K		Sk/23 144N		Sk/26 45N/144N	
Antigens	HI	Neutralization	HI	Neutralization	HI	Neutralization
A/Okayama/2/11 (Oka/2 144K)	*160*	*270*	320	270	320	405
A/Shizuoka/23/12 (Sk/23 144N)	40	90	*160*	*90*	320	270
A/Shizuoka/26/12 (Sk/26 45N/144N)	80	90	320	90	*640*	*270*

*Italics*: hemagglutination inhibition and neutralization titer for homologous strains

Neutralization data are shown as the mean value of four independent experiments

### Effect of amino acid substitutions at position 144 of the HA globular head on viral growth, as evaluated by the plaque reduction neutralization test

**Fig. 3:**
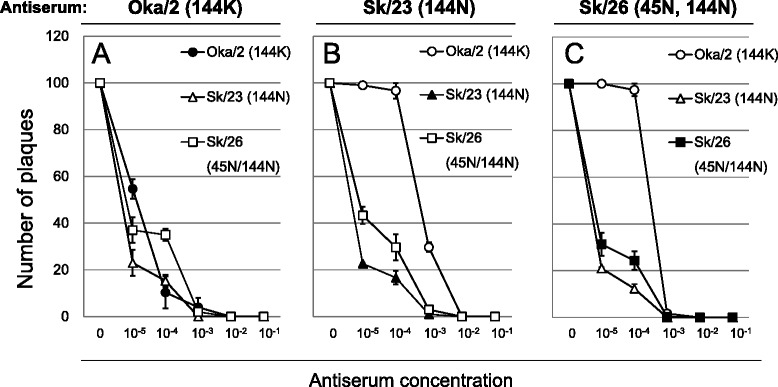
Neutralizing effects of guinea pig antiserum against homologous and heterologous viruses. The property of the guinea pig antisera raised against three types of the clinical viral isolates at a limiting concentration. Oka/2, Sk/23, and Sk/26 were used as representative 144K, 144N, and 45N/144N type viruses, respectively, to obtain neutralizing antibodies. **a** The neutralizing activity of anti-Oka/2 (144K) antiserum against Oka/2 (144K), Sk/23 (144N) and Sk/26 (45N/144N) viruses. **b** The neutralizing activity of anti-Sk/23 (144N) antiserum against Oka/2 (144K), Sk/23 (144N) and Sk/26 (45N/144N) viruses. **c**. The neutralizing activity of anti- Sk/26 (45N/144N) antiserum against Oka/2 (144K), Sk/23 (144N) and Sk/26 (45N/144N) viruses. Results are presented as means ± SD of three independent experiments with duplicate wells

To understand the potential mechanism underlying the variations in N- glycosylation-associated amino acid residues in the HA of H3N2 viruses (Fig. 2), we further tested the property of the guinea pig antisera raised against the three types of the clinical viral isolates at a limiting concentration, which mimics the low antibody titers in patients after a certain period of time from the previous exposure. The virus concentration was set to 100 plaque forming units, and the neutralization function of the antisera on viral growth was evaluated by plaque reduction neutralization assays as previously described [15] (Fig. 3). As shown in Fig. 3, the number of plaques produced by the Oka/2 (144K) virus was reduced by 90 % when the viral suspension was pre-treated with 1:10000 diluted anti-Oka/2 (144K) antiserum (homologous combination), indicating strong neutralizing activity (Fig. 3a). The neutralizing activity of anti-Oka/2 (144K) antiserum against the Sk/23 (144N) and Sk/26 (45N/144N) viruses (heterologous combination) was 85 % and 65 %, respectively, indicating substantial cross-reactive neutralizing activity of anti-Oka/2 (144K) antiserum against these viruses (Fig. 3a). The neutralization activity of anti-Sk/23 (144N) antiserum against Sk/23 (144N) virus (homologous pair) and anti-Sk/23 (144N) antiserum against Sk/26 (45N/144N) virus (heterologous pair) was sufficiently strong (Fig. 3b). Similarly, the neutralization activity of anti-Sk/26 (45N/144N) antiserum against Sk/26 (45N/144N) virus (homologous pair) and anti-Sk/26 (45N/144N) antiserum against Sk/23 (144N) virus (heterologous pair) was also sufficiently strong (Fig. 3C). However, the neutralization activity of 1:10000 diluted anti-Sk/23 (144N) and anti-Sk/26 (45N/144N) antisera against the Oka/2 (144K) virus was only 3.3 % and 2.7 %, respectively (Fig. 3b, c). These results indicate that the antisera against the 2011–12 season viruses (i.e., Sk/23 and Sk/26) do not effectively neutralize the 2010–11 season virus (Oka/2) at a limiting concentration.

### The NGS at residue 144 in the HA globular head of H3N2 significantly affected antibody mediated neutralization

**Fig. 4:**
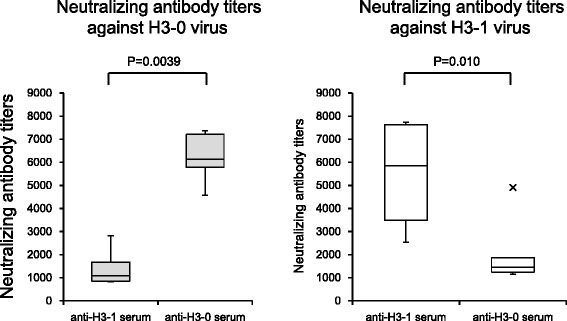
Cellular immune response to different influenza A (H3N2) virus strains with or without NGS at residue 144 in the globular head of HA. Humoral immune response to different influenza A (H3N2) virus strains with or without NGS at residue 144 in the globular head of HA evaluated by reverse genetics approach. The neutralizing ability of serum samples were evaluated by plaque reduction neutralization test. Statistical differences were calculated using a Mann–Whitney *U* test

Finally, to examine whether the NGS at residue 144 of HA affected antibody mediated neutralization, we generated mutant viruses with or without the NGS at residue 144 in the HA by reverse genetics. The cDNA of the HA gene of A/Okayama/6/01 (H3N2) was generated by RT-PCR, and two mutant viruses were produced; virus harboring NGS at residue 144 in the HA (code-named H3-0) and virus lacking NGS at residue 144 in the HA (code-named H3-1). H3-0 virus has seven potential NGSs, whereas H3-1 virus has six potential NGSs (Table 4). Two groups of six mice each were intra nasally infected with 10^5^ PFU of H3-0 or H3-1 viruses in 20 μl of PBS. Four weeks after infection, the mice were sacrificed, and the serum neutralizing antibody titers against H3-0 or H3-1 viruses were determined by the plaque reduction neutralization test. As shown in Fig. 4, we did not observe effective neutralization of the H3-1 virus by the antiserum raised against H3-0 virus, and vice versa.

**Table 4:** Mutant H3N2 viruses with or without asparagine (N)-linked glycosylation site at residue 144 in hemagglutinin

Virus	Glycosylation sites in the HA globular head						
	63	122	126	133	144	165	246
H3-0 (7 NGSs)	NCT	NES	NWT	NGT	NKS	NVT	NCT
H3-1 (6 NGSs)	NCT	NES	NWT	NGT	NKA	NVT	NCT

Residues 63, 122, 126, 133, 144, 165, and 246 are located in the globular head of hemagglutinin (HA)

H3-0 virus has seven potential asparagine (N)-linked glycosylation sites (NGSs) in the HA globular head

H3-1 is the mutant virus lacking NGS in the HA globular head at position 144; the mutation is produced by introducing a single amino acid substitution, namely, Ser (S) to Ala (A), at residue 146 of H3-0. Thus, H3-1 virus has six potential NGSs in the HA globular head

## Discussion

IAV escapes host immune response by changing the antigenicity of HA and neuraminidase, both gradually (antigenic drift) and abruptly (antigenic shift) [16]. Antigenic drift is achieved via changes in the amino acids at the antigenic sites that are recognized by antibodies [17–19]. Since the efficacy of vaccines requires a close antigenic match between circulating and vaccine strains, and since mismatches result in increased disease burden, it is important to identify the mutations that affect significantly the neutralizing antibody response mounted against natural infection or vaccination. Especially, the antigenic sites of HA, which affect the receptor binding properties [20] and virulence [11] are crucial for understanding antigenic drift and vaccine strain selection. Moreover, it has been proposed that the attachment of a glycosyl chain to an NGS (Asn-X-Ser/Thr, where X is any amino acid except Pro) in the globular head of HA contributes to immune escape [7, 20–24].

In the present study, we observed that H3N2 became predominant in the seasons (2010–11 to 2011–12) immediately following the 2009 pandemic of H1N1, and almost all the isolates had an N residue that forms an additional NGS at residue 144 of HA in the 2011–12 season. Interestingly, there was an antigenic mismatch between the vaccine strain and the circulating viruses in the 2011–12 season. The vaccine strain used in the 2011–12 season had a K residue that cannot form NGS at position 144 of HA (Table 5 and Fig. 2). It may therefore be possible that the newly acquired NGS in the HA globular head is associated with the re-emergence of H3N2 through escape from humoral immunity within this subtype. There was re-emergence of H1N1 in the 2013–14 season in both Japan and United States (Fig. 1). However, the virus characterization data indicated that the re-emerged H1N1 was genetically and antigenically similar to H1N1pdm09 [25]. Therefore, antigenic drift did not play an important role in the re-emergence. Other factors such as a waning of immunity in the population or vaccine effectiveness may be responsible for this re-emergence.

**Table 5:** The strains used in seasonal influenza vaccines from 2001 to 2015 and amino acid residue(s) of potential N-linked glycosylation sites on the globular head of HA in these strains

Year	Northern hemisphere	Amino acid residue(s)		Southern hemisphere	Amino acid residue(s)	
		144	144-146		144	144-146
2014–15	A/Texas/50/2012 (H3N2)	N	NNS	A/Switzerland/9715293/2013 (H3N2)	N	NSS
2013–14	A/Victoria/361/2011(H3N2)	N	NNS	A/Texas/50/2012 (H3N2)	N	NNS
2012–13	A/Victoria/361/2011(H3N2)	N	NNS	A/Victoria/361/2011(H3N2)	N	NNS
2011–12	A/Perth/16/2009 (H3N2)	K	KNS	A/Perth/16/2009 (H3N2)	K	KNS
2010–11	A/Perth/16/2009 (H3N2)	K	KNS	A/Perth/16/2009 (H3N2)	K	KNS
2009–10	A/Brisbane/10/2007 (H3N2)	N	NNS	A/Perth/16/2009 (H3N2)	K	KNS
2008–09	A/Brisbane/10/2007 (H3N2)	N	NNS	A/Brisbane/10/2007 (H3N2)	N	NNS
2007–08	A/Wisconsin/67/2005 (H3N2)	N	NNS	A/Brisbane/10/2007 (H3N2)	N	NNS
2006–07	A/Wisconsin/67/2005 (H3N2)	N	NNS	A/Wisconsin/67/2005 (H3N2)	N	NNS
2005–06	A/California/7/2004(H3N2)	N	NNS	A/California/7/2004(H3N2)	N	NNS
2004–05	A/Fujian/411/2002(H3N2)	N	NKS	A/Wellington/1/2004(H3N2)	N	NKS
2003–04	A/Moscow/10/99(H3N2)	I	INS	A/Fujian/411/2002(H3N2)	N	NKS
2002–03	A/Moscow/10/99(H3N2)	I	INS	A/Moscow/10/99(H3N2)	I	INS
2001–02	A/Moscow/10/99(H3N2)	I	INS	A/Moscow/10/99(H3N2)	I	INS

We tested the effect of amino acid substitutions on the neutralizing activity of guinea pig antisera raised against H3N2 strains with or without additional NGSs. As expected, the results of both HI and microneutralization assays showed that the anti-Oka/2 (144K) serum showed lower neutralizing activity against both Sk/23 (144N) and Sk/26 (144N/45N) viruses compared to that of anti-Sk/23 and anti-Sk/26 sera against the Oka/2 (144K) virus; this suggests that substitution of the amino acid residue at position 144 might modulate the conformational fit of the antibody to the antigenic site of HA as a means of immune escape. However, in clear contrast, the plaque reduction neutralization assay showed that anti-Oka/2 serum can block the mutated Sk/23 and Sk/26 virus strains in a slightly better manner than the Oka/2 virus itself even at the lowest concentration (1:10000 dilution), whereas Oka/2 (144K) virus was barely neutralized with the anti-Sk/23 (144N) and anti-Sk/26 (144N/45N) sera. Perhaps most important information from this result is that the plaques can be detected at lower antiserum concentration. Namely, the antibody-mediated immune evasion becomes invalid at a “limiting concentration”, which mimics low antibody titers in patients after a certain period from the previous exposure or low vaccine effectiveness. If this is the case, added glycosylation site to a virus does not allow it to escape antibody directed against a less glycosylated variant, but a less glycosylated virus can escape immunity mounted against a more glycosylated variant. This may explain the observed fluctuations (i.e., gains and losses) in the N-glycosylation associated amino acid residue 144 in the HA globular head (Fig. 2). Indeed, vaccine viruses used during the 2004–2007 seasons contained 144N (Table 5), which might have resulted in the emergence of 144K mutant, and then some natural viral fitness issue could have caused it to disappear again. It is conceivable that not only gains but also losses of N-linked glycan at the amino acid 144 of HA are effective strategies for persistence of circulating H3N2 virus even in the face of the humoral immune response. It is, of course, possible that these fluctuations are due to the non-neutralizing function of the antisera against emerged viruses at a limiting concentration. In other words, the influenza virus can survive at sufficiently low titers of antibody against the virus load in some cases.

To exclude the possibility that the observed neutralizing activity of serum is not due to the newly acquired N-glycosylation in the HA globular head, we generated mutant viruses with and without this particular NGS at residue 144 in the HA by a reverse genetics approach, and tested whether the substitution of the amino acid residue 144 significantly affected the neutralization activity. As expected, the neutralizing ability against a heterologous H3N2 strain was significantly reduced when the NGS at residue 144 was replaced, suggesting that the newly acquired NGS at residue 144 in the HA globular head may play an important role in the re-emergence of the endemic seasonal H3N2 within the subtype by helping it evade the humoral immunity. It is noteworthy that previous studies have also suggested the importance of amino acid changes in this region, entitled antigenic site A, for the generation of antigenically distinct viruses of epidemic significance [26]. For instance, substitutions at the predicted NGS at position 144 in HA have been shown to contribute to increased infectivity of the reassorted H3N2 viruses of the 2003–2004 season, causing an epidemic in Denmark [27]. Interestingly, our data showed that the substitution at residue 45 in the stem region of HA also had some effect on the plaque reduction neutralization assay.

As mentioned above, we found a new amino acid substitution at residue 45 of the stem of HA in 2011–12 season. Interestingly, not only in Japan, but also in Tunisia, 45N and 144N type H3N2 viruses (same as Sk/26 in this study) have been detected in one severe and one fatal case, whereas 45S and 144D type viruses were detected in one severe case and two mild cases, in 2013 [28]. Since the stem of HA has been assumed to provide the main forces that stabilize HA trimer [29], it is well known that the stem region of the viral HA tends to be conserved across different virus strains, whereas the globular head region shows considerable variation [30]. Thus, the conserved stem domain of HA is considered to be a potential “universal” vaccine candidate with the potential to confer heterosubtypic protection. It has been reported that in H3N2 viruses, there are two NGSs in the stem of HA (nos. 22 and 38) [30]. These two residues were indeed conserved in our samples throughout the study period, whereas a new amino acid substitution at residue 45 of the stem of HA was found in the 2011–12 season. It seems likely that although the addition of N-linked glycans may contribute to viral escape from neutralizing antibodies, the addition of glycans may also interfere with the receptor binding properties of HA, resulting in lower levels of viral entry and infection. It is therefore possible that the amino acid substitution observed at the NGS site within the stem of HA is a result of compensation, which increases binding affinity for efficient infection and viral replication. Further research is needed to confirm this hypothesis.

## Conclusions

Our results suggest that occurrence of immune-escape mutations at residue 144 in HA is an important determinant for the re-emergence of H3N2 within the subtype after the 2009 pandemic. The findings of this study may also contribute to enhancing the understanding of the genetic evolution of H3N2 viruses. The ongoing monitoring of genetic drift, especially in the HA of circulating H3N2 virus, may be of fundamental importance for vaccine design.

## Abbreviations

HA, hemagglutinin; HI, hemagglutination inhibition; IAVs, influenza A viruses; NGSs, asparagine (N)-linked glycosylation sites
